# Lipocalin 2 Protects Against *Escherichia coli* Infection by Modulating Neutrophil and Macrophage Function

**DOI:** 10.3389/fimmu.2019.02594

**Published:** 2019-11-08

**Authors:** Qianqian Wang, Shuhui Li, Xueyou Tang, Li Liang, Fengqin Wang, Huahua Du

**Affiliations:** MoE Key Laboratory of Molecular Animal Nutrition, College of Animal Sciences, Zhejiang University, Hangzhou, China

**Keywords:** lipocalin 2, Lcn2-deficient mice, bacterial challenge, neutrophil, macrophage

## Abstract

Lipocalin 2 (Lcn2) is an essential component of the antimicrobial innate immune system. It attenuates bacterial growth by binding and sequestering the iron-scavenging siderophores to prevent bacterial iron acquisition. Whereas, the ability of Lcn2 to sequester iron is well-described, the role of Lcn2 in regulating immune cells during bacterial infection remains unclear. In this study, we showed that upon infection with *Escherichia coli* (O157:H7), Lcn2-deficient (*Lcn2*^−/−^) mice carried more bacteria in blood and liver, and the acute-phase sera lost their antibacterial activity *in vitro*. Neutrophils from *Lcn2*^−/−^ mice were defective in homeostasis and morphological development. *E. coli* O157:H7 infection of *Lcn2*^−/−^ mice resulted in a reduced neutrophil migration capacity, with 30% reduction of extravasated neutrophils, and impaired chemotaxis, as shown by a reduction in the secretion of chemoattractants, such as tumor necrosis factor (TNF)-α, monocyte chemoattractant protein (MCP)-1, and macrophage inflammatory protein (MIP)-2, which are instrumental in eliciting a neutrophil response. We also found that some secreted cytokines [interleukin (IL)-6, IL-1β, and TNF-α] were decreased. Transcripts of inflammatory cytokines (IL-6, IL-1β, TNF-α, and IL-10), chemokines (MIP-2 and MCP-1), and iNOS production were all strongly repressed in *Lcn2*^−/−^ macrophages. Furthermore, Lcn2 could induce the production of chemokines and promote the migration and phagocytosis of macrophages. Thus, Lcn2 deficiency could impair the migration and chemotaxis ability of neutrophils and disturb the normal secretion of inflammatory cytokines of macrophages. Therefore, the heightened sensitivity of *Lcn2*^−/−^ mice to *E. coli* O157:H7 is not only due to the antibacterial function of Lcn2 but also a consequence of impaired functions of immune cells, including neutrophils and macrophages.

## Introduction

Iron is an essential micronutrient required for almost all aerobic organisms, with crucial functions in many critical metabolic processes, such as DNA synthesis, oxygen transport, redox reaction, and synthesis of hemoglobin (1). Both hosts and pathogens depend on and compete for iron for their proliferation and biologic functions. Therefore, iron always lies at the center of an eons-long battle between hosts and their pathogens (2). In the struggle for iron, bacteria have evolved aggressive iron-acquiring mechanisms through the expression of siderophores to steal iron from host proteins, such as transferrin and ferritin (3). As a leading bacteria that cause diarrhea in humans and livestock animals, *Escherichia coli* can detect low iron signal as an environmental cue to trigger the synthesis of siderophore enterobactin, which has high affinity for iron (4, 5). In order to restrict bacteria from obtaining iron, the hosts have also adopted some “nutritional immunity” mechanisms for the competition of iron, including lipocalin 2 (Lcn2) (6). Lcn2 has higher affinity to enterobactin-Fe^3+^ than enterobactin receptor protein FepA of *E. coli*, so it can inhibit the iron uptake pathway of *E. coli* and disrupt bacterial iron acquisition (7).

Lcn2, also known as neutrophil gelatinase-associated lipocalin (NGAL), siderocalin, or 24p3, is a multipotent 25-kDa protein and mainly secreted by neutrophils. As a member of the lipocalin superfamily, Lcn2 forms a barrel-shaped tertiary structure with a hydrophobic calyx that binds several lipophilic molecules (8). It is a pleiotropic mediator of various biochemical processes, such as iron delivery (9), apoptosis (10), and cell migration and differentiation (11). Lcn2 also plays an important role as an early marker for kidney damage (12). Of all those functions, the best characterized one is that Lcn2 obstructs the siderophore iron-acquiring strategy of bacteria and thus inhibits bacterial growth. Indeed, Lcn2-deficient (*Lcn2*^−/−^) mice were more sensitive to bacterial infection than wild-type (WT) mice and exhibit higher mortality rates after systemic administration of *E. coli* (13, 14). In this regard, Lcn2 plays an essential role in the innate immune response against bacterial infection.

Despite being named as a neutrophil protein and originally identified as a component of neutrophil granules, Lcn2 can also be expressed in other cell types, including macrophages, hepatocytes, epithelia, and adipocytes (13, 15, 16). Lcn2 has been reported to be an acute-phase protein based on elevated levels in serum, epithelium, urine, and feces of patients with active inflammatory disease (17–19). However, the precise role of Lcn2 in bacterial infection remains to be elucidated. Therefore, in this study, we investigated the role of Lcn2 in *E. coli* O157:H7 infection using gene-targeted *Lcn2*^−/−^ mice. Our results present evidence to show that Lcn2 was dramatically upregulated and mainly induced in the liver in challenged mice. We showed that *Lcn2*^−/−^ mice exhibited increased susceptibility to bacterial infections, in keeping with the proposed function of Lcn2 in iron sequestration. Moreover, we found that neutrophils derived from *Lcn2*^−/−^ mice were defective in homeostasis, morphology, and migration. Additionally, Lcn2 was necessary for macrophages to induce inflammatory cytokines and phagocytose bacteria. Therefore, the observed sensitivity of *Lcn2*^−/−^ mice to the pathogen *E. coli* O157:H7 is not only related to the antibacterial function of Lcn2 resulting from sequestration of iron but also a consequence of impaired immune cell function, such as neutrophils and macrophages.

## Materials and Methods

### Mice and Cell Culture

C57BL/6 WT and C57BL/6 Lcn2-deficient (*Lcn2*^−/−^) male mice (~20 g) were obtained from Animal Center of Chinese Academy of Sciences (Shanghai, China) and Jackson Laboratory (USA), respectively. All mice were housed in specific pathogen-free cages and received food and water *ad libitum* in Zhejiang University with a 12-h dark-light cycle at 24°C. No mouse died during the experiment. Mouse studies were approved by the Animal Ethics Committee of Zhejiang University.

RAW264.7 macrophages were obtained from the Cell Bank of the Chinese Academy of Sciences (Shanghai, China) and maintained in RPMI-1640 (Gibco, USA) supplemented with 10% fetal bovine serum (FBS) (Gibco, USA), penicillin (KeyGen Biotech, China) (100 IU/ml), and streptomycin (KeyGen Biotech, China) (100 μg/ml) at 37°C in humidified air containing 5% CO_2_. Cells were seeded in six-well dishes at 1 × 10^6^ cells per well and grown overnight until 80% confluent. They were then digested by EDTA-trypsin (KeyGen Biotech, China) and used for a variety of experimental procedures.

### *E. coli in vitro* Infection

*E. coli* O157:H7 (ATCC43889 strain) was obtained from China General Microbiological Culture Collection Center (Beijing, China) and grown in Luria-Bertani (LB). Prior to *in vitro* infection, cells were extensively washed with phosphate-buffered saline (PBS) (Genome Biotech, China) and incubated in complete RPMI-1640 without antibiotics for 2~3 h until 90% confluent. The concentration of bacteria solution was determined by a standardized calibration curve of OD600/colony-forming units (CFU).

### *E. coli in vivo* Infection

Each mouse (5–6 weeks) was infected by intragastric administration with 2 × 10^8^ CFU of *E. coli* O157:H7 diluted in 200 μl PBS. Mouse behavior was carefully monitored every 12 h. For investigating the expression changes of Lcn2 after bacterial challenge, a total of 32 mice (*n* = 4 per time point) were euthanized by cervical dislocation at 1, 4, 8, 24, 32, 36, 48, and 60 h post infection (hpi). Liver, spleen, kidney, jejunum, colon, lung, and heart were collected for quantitative real-time PCR detection. For measuring the bacterial burden, heparinized blood samples and homogenized liver were collected at 32 hpi and plated on LB agar to determine CFU. Blood samples were also used for Wright-Giemsa staining (Phygene, China) and measurements of serum Lcn2 protein. Liver and jejunum tissues were fixed for paraffin sectioning and immunohistochemistry (frozen sections). Uninfected control group (*n* = 4) received 200 μl of sterile solution containing 10% (w/v) NaHCO_3_ and 20% (w/v) sucrose.

For determining the bacteriostatic ability of endogenous Lcn2, WT and *Lcn2*^−/−^ mice (*n* = 6 per group) were intraperitoneally injected with 2 × 10^8^ CFU heat-killed *E. coli* O157:H7. Acute-phase serum was collected at 5 hpi. *E. coli* O157:H7 (10^3^ CFU) were then inoculated into RPMI-1640 with 20% acute-phase serum from WT and *Lcn2*^−/−^ mice. The heat-killed *E. coli* O157:H7 solution was produced by heating in a water bath at 100°C for 30 min.

### Determination of Neutrophils in Peripheral Blood and Peritoneal Exudates

Mice were intraperitoneally injected with 1 ml 2 × 10^8^ CFU heat-killed *E. coli* O157: H7 to induce peritonitis (*n* = 6 per group). Mice were sacrificed at 5 hpi. The peripheral blood was drawn from retroorbital plexus, and the peritoneal exudates were extracted from the peritoneal cavity. Neutrophils from peripheral blood and peritoneal exudates were labeled by the phycoerythrin (PE)-conjugated rat anti-mouse Ly6G (Gr-1) mAb (clone 1A8) (BD Biosciences, USA). The percentage of neutrophils was determined by flow cytometry analysis.

### Immunohistochemical Assay

Tissues sections from liver and jejunum were deparaffinized with xylene and rehydrated through a series of graded alcohol solutions to deactivate endogenous enzymes. Then, they were washed with PBS and immersed in 0.01M citric acid buffer at 98–100°C to reveal antigens. Cooled sections were stained using a goat anti-mouse Lcn2 (1:250; R&D, USA) and appropriate horseradish peroxidase (HRP)-conjugated secondary antibodies (1:1,000; Pierce, USA).

### ELISA

Serum Lcn2 was quantified using Lcn2 Mouse ELISA kits (Boster, China) according to the manufacturer's instructions. Cytokine tumor necrosis factor α (TNF-α levels in serum and peritoneal lavages were measured by Duoset ELISA cytokine kits (Rapidbio, USA) according to the manufacturer's instructions. Interleukin (IL)-6, IL-1β, TNF-α, monocyte chemoattractant protein (MCP)-1, and macrophage inflammatory protein (MIP)-2 levels in the supernatants of the culture medium were quantified using Mouse ELISA kits (eBioscience, USA) according to the manufacturer's instructions.

### Bone Marrow-Derived Macrophage (BMDM) Isolation and Culture

BMDMs were isolated from the cavity of femur and tibia of WT and *Lcn2*^−/−^ mice (*n* = 6 per group) after removing the attached muscle tissues. They were then cultured in six-well plates with complete DMEM medium containing 20% fetal bovine serum (FBS), 1% penicillin and streptomycin, and 30% conditioned L929 media as a source of macrophage colony-stimulating factor (M-CSF). On day 7, BMDM cultures with nearly 100% confluence were stimulated with 5 × 10^6^ CFU *E. coli* O157:H7 in DMEM for 24 h. Cytokines were analyzed in the supernatants.

### RNA Extraction and Quantitative Real-Time PCR

Total RNA was extracted from cells and animal tissues by the Trizol method (Sigma, China). For determining the tissue specificity of Lcn2 gene expression, kidney, pancreas, heart, lung, spleen, liver, jejunum, colon, adipose, bone, muscle, testis, and brain were collected for RNA extraction. Reverse transcription was performed on 2 μg of RNA using random hexamers and reverse transcriptase (Thermo-Fisher Scientific, USA). Quantitative real-time PCR was performed using the FastStart Universal SYBR Green Master fluorescence quantitative kit (Roche, Switzerland). All data were normalized to a housekeeping gene glyceraldehyde-3-phosphate dehydrogenase (GAPDH) or β-actin measured in the same sample. The sequences of the specific primers are listed in Table 1. The fold change was calculated using ΔΔ threshold cycle method.

**Table 1:** Primer sequences for the real-time PCR amplification.

**Gene**	**Primer (5^′^ → 3^′^)**	**Genebank no**.
β-actin	F: CCACCATGTACCCAGGCATT	NM_007393.5
	R: AGGGTGTAAAACGCAGCTCA	
IL-1β	F: AGTTGACGGACCCCAAAAG	NM_008361.4
	R: TTTGAAGCTGGATGCTCTCAT	
IL-10	F: TGGGTTGCCAAGCCTTATCG	NM_010548.2
	R: TTCAGCTTCTCACCCAGGGA	
GAPDH	F: TGCGACTTCAACAGCAACTC	NM_008084.3
	R: GCCTCTCTTGCTCAGTGTCC	
MCP-1	F: GATGCAGTTAACGCCCCACT	NM_011333.3
	R: ACCCATTCCTTCTTGGGGTC	
TNF-α	F: GCTCTTCTGTCTACTGAACTTCGG	NM_013693.3
	R: ATGATCTGAGTGTGAGGGTCTGG	
MIP-2	F: CACTCTCAAGGGCGGTCAAA	NM_009140.2
	R: GGTTCTTCCGTTGAGGGACA	
IL-6	F: CCCCAATTTCCAATGCTCTCC	NM_031168.2
	R: CGCACTAGGTTTGCCGAGT	
GM-CSF	F: GCCATCAAAGAAGCCCTGAAC	NM_009969.4
	R: TCTTCAGGCGGGTCTGCAC	
iNOS	F: CTCACCTACTTCCTGGACATTAC	NM_010927.4
	R: CAATCTCTGCCTATCCGTCTC	
Lcn2	F: ACATTTGTTCCAAGCTCCAGGGC	NM_008491.1
	R: CATGGCGAACTGGTTGTAGTCCG	

### Analysis of Peripheral Blood Smears

A drop of peripheral blood was smeared onto a clean glass side and quickly air-dried for 30 min at room temperature. The smears were then stained with Diff-Quick staining kit (Dade-Behring) according to the manufacturer's recommended protocols. Representative images were shown.

### Scratch Wound Healing Assay

Cell migration was examined using the scratch wound healing assay with RAW264.7 macrophages (20). RAW264.7 macrophages were seeded in six-well plates at a density of 2 × 10^3^ cells/well and incubated overnight until cells were ~70% confluent as a monolayer. The monolayer of cells was gently and slowly scratched linearly with a sterile 10-μl pipette tip to create a wound. Cells were washed twice with PBS to remove floating cells and treated with 1 μg/ml recombined Lcn2 protein (Abcam, USA), while control groups were left untreated. The cells migrating from the leading edge were imaged at 0 (immediately after scratching) and 24 h. The migration distance was calculated by subtraction of the gap distance from the same point at 0 and 24 h.

### Phagocytosis Analysis

After infection, the cells were incubated with serum-free medium containing 0.5 mg/ml fluorescein isothiocyanate (FITC)-dextran (4 kDa) (Sigma, America) for 2 h. Extracellular FITC-dextran was washed away with PBS. The cells were dissociated from the cell culture dishes (Corning, USA) with EDTA-trypsin solution (KeyGen Biotech, China), and the intracellular FITC fluorescence intensity was measured by flow cytometry.

### Immunofluorescence Analysis for iNOS Determination

RAW264.7 macrophages seeded on glass-bottom dishes were fixed in 4% paraformaldehyde (KeyGen Biotech, China) and then permeabilized with ice-cold 0.5% TritonX-100. The cells were blocked in PBS containing 10% bovine serum albumin (KeyGen Biotech, China) for 30 min and then were incubated with rabbit monoclonal antibody inducible nitric oxide synthase (iNOS) (Bioss Antibodies, China) and rat monoclonal antibody F4/80 (Abcam, USA) overnight at 4°C. After washing with PBS, cells were incubated with Alexa Fluor 488 goat anti-rabbit IgG (Abcam, USA) and Alexa Fluor 647 goat anti-rat IgG (Abcam, USA) for 1 h at 37°C. Finally, cells were counterstained with 50 mg/ml 4′,6-diamidino-2-phenylindole (DAPI) (KeyGen Biotech, China) before capturing images with a confocal microscope (Zeiss, Germany).

### Statistical Analysis

Statistical analysis of experimental data was performed by Student's *t*-test and one-way ANOVA in SPSS 20.0 software. The results are expressed as mean ± SEM. The test results were independently repeated three or six times. Levels of statistical significance were set at *P* < 0.05.

## Results

### Lcn2 Increases Dramatically During *E. coli* O157:H7 Infection

**Figure 1:**
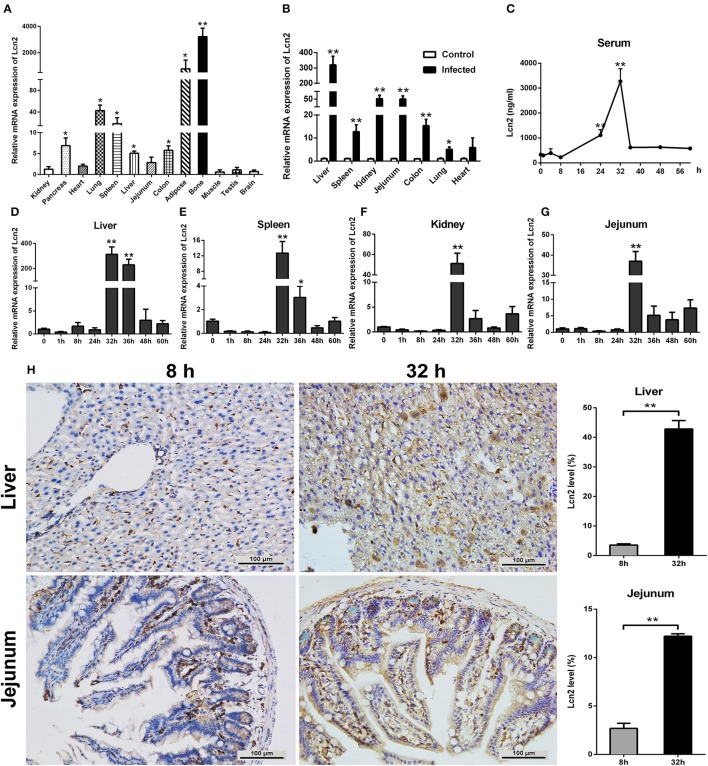
Elevated lipocalin 2 (Lcn2) during *E. coli* O157:H7 infection. **(A)** Real-time PCR analysis of the levels of Lcn2 mRNA expression in indicated tissues of mice. **(B)** The mRNA levels of Lcn2 expression in indicated tissues of control and *E. coli* O157:H7-infected mice. **(C)** Serum levels of Lcn2 protein concentration in *E. coli* O157:H7-infected mice after challenge at different time points. **(D–G)** The mRNA expression levels of Lcn2 in indicated tissues from *E. coli* O157:H7-infected mice after challenge at different time points. **(H)** Protein levels of Lcn2 in mice detected on immunohistochemistry sections of liver and jejunum at 8 and 32 h after challenge with *E. coli* O157:H7. Original magnification was 200×. Values are average means of triplicate experiments. Error bars depict SEM (*n* = 4 per time point). Results are expressed as means ± SEM. *P < 0.05, and **P < 0.01.

In order to determine the tissue specificity of Lcn2 gene expression, Lcn2 mRNA expression was examined by qRT-PCR in most tissues of mice. Lcn2 mRNA was mainly expressed in bone marrow, adipose, lung, and spleen, while it was less expressed in muscle, testis, and brain (Figure 1A). The transcript of Lcn2 was highest in the bone marrow, which was more than 3,000 times of that in the kidney. To test the hypothesis that Lcn2 is one of the acute-phase proteins, we determined Lcn2 expression of challenged mice by intragastric administration with a sublethal dose of a clinical strain of *E. coli* O157:H7 (Figure S1). Thirty-two hours after challenge, Lcn2 mRNA levels were markedly (*P* < 0.01) increased in the liver, jejunum, kidney, colon, and spleen (Figure 1B). The ratio of Lcn2 mRNA in the liver of infected mice to that of control mice was about 300. Thus, it seems that Lcn2 expression is mainly induced in the liver during *E. coli* O157:H7 infection. To investigate the effect of bacterial infection on the level of Lcn2 in the bloodstream, sera were collected, and Lcn2 was detected by ELISA. The basal serum concentration of Lcn2 in uninfected mice was ~300 ng/ml (Figure 1C). After intragastric infection, serum levels of Lcn2 increased to 1,110 ng/ml by 24 h, peaked at 3,270 ng/ml by 32 h, and then rapidly declined (Figure 1C). This expression profile of Lcn2 protein was in concordance with observations of Lcn2 transcripts in detected tissues, including the liver (Figure 1D), spleen (Figure 1E), kidney (Figure 1F), and jejunum (Figure 1G). Furthermore, immunohistochemical assay was employed to detect the Lcn2 protein in the liver and jejunum. *E. coli* O157:H7 infection induced a 12-fold increase of Lcn2 in the liver and a five-fold increase in the jejunum (Figure 1H). Thus, infection with *E. coli* O157:H7 elevated the levels of both Lcn2 mRNA and protein in the tissues and bloodstream of mice.

### Lcn2 Is Involved in the Antibacterial Responses to *E. coli* O157:H7 Infection

**Figure 2:**
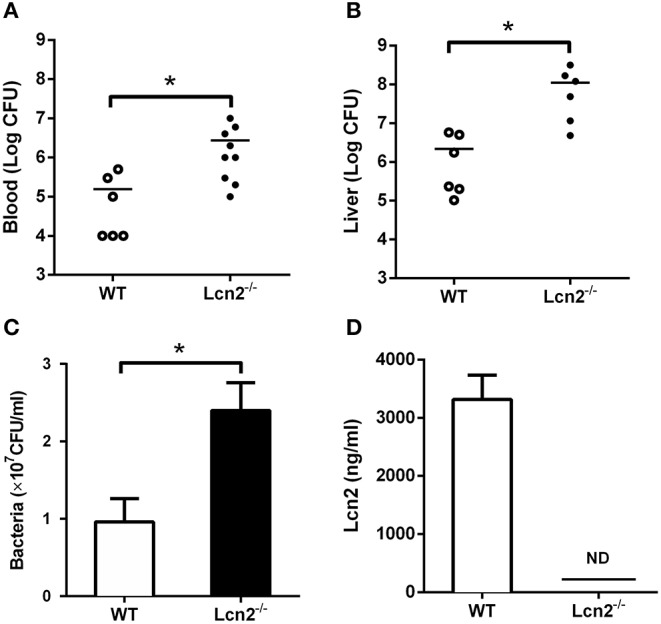
The bacteriostatic characteristics of lipocalin 2 (Lcn2). **(A,B)** Bacterial loads in blood (CFU/ml) and livers (CFU/mg) of *E. coli* O157:H7-infected mice 32 hpi. **(C,D)** Serum levels of Lcn2 protein measured and growth of *E. coli* O157:H7 in RPMI with 20% acute-phase serum from wild-type (WT) or *Lcn2*^−/−^ mice. Values are average means of triplicate experiments with two mice per genotype per experiment. Error bars depict SEM (*n* = 6 per group). Results are expressed as means ± SEM. *P* < 0.05 was considered statistically significant. *P < 0.05.

Since *E. coli* O157:H7 challenge induced increased Lcn2 levels of transcription and protein in tissues and blood of mice, we speculated that Lcn2 might play a role in responses against *E. coli* O157:H7 infection. To test the role of Lcn2 in an acute lethal infection, we challenged *Lcn2*^−/−^ or WT mice by intragastric administration with 2 × 10^8^ CFU of *E. coli* O157:H7 and measured the bacterial burden. Compared with the titer in control WT mice, the bacteria recovered from *Lcn2*^−/−^ mice were ~20-fold higher in blood, where there are 2.7 × 10^6^ CFU/mg in *Lcn2*^−/−^ mice and 1.5 × 10^5^ CFU/mg in WT mice (Figure 2A). In the liver, there were 1.2 × 10^8^ CFU/mg in *Lcn2*^−/−^ mice and 2.1 × 10^6^ CFU/mg in WT mice. The bacteria from *Lcn2*^−/−^ mice were ~60-fold higher (Figure 2B). In order to determine the direct bacteriostatic activity of Lcn2, sera from *Lcn2*^−/−^ mice inoculated by heat-killed *E. coli* O157:H7 were collected 5 hpi and used for bacterial incubation. Compared with *Lcn2*^−/−^ mice, the serum from WT mice exerted remarkable inhibition to *E. coli* O157:H7 (*P* < 0.05) (Figure 2C). Meanwhile, there were more than 3,000 ng/ml Lcn2 proteins in the serum of WT mice, but no Lcn2 was detected in serum of *Lcn2*^−/−^ mice as expected (Figure 2D).

### Lcn2 Deficiency Alters Neutrophil Homeostasis

**Figure 3:**
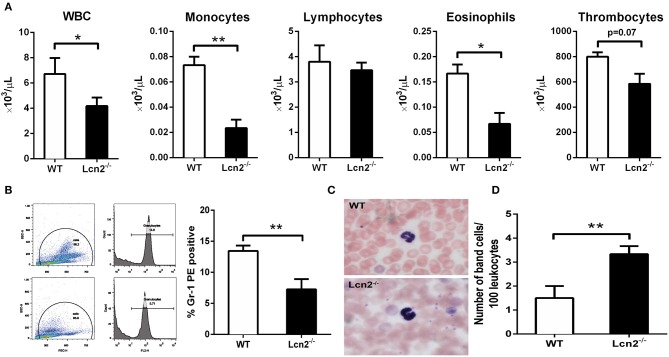
Granulocyte abnormalities in *Lcn2*^−/−^ mice. **(A)** Hematological parameters of peripheral blood from wild-type (WT) and *Lcn2*^−/−^ mice. The data are presented as mean × 10^3^ cells/μl. Error bars depict SEM (*n* = 6 per group). **(B)** Flow cytometry analysis of neutrophils in the peripheral blood after intragastric administration with 2 × 10^8^ CFU of *E. coli* O157:H7. Cells were stained with indicated clones of Gr-1 Ab, and positive cells were determined by flow cytometry. Values are average means of triplicate experiments with two mice per genotype per experiment. Error bars depict SEM. **(C)** Wright-Giemsa staining of peripheral blood smears from *Lcn2*^−/−^ mice identified atypical hyposegmented neutrophils in the peripheral blood. Original magnification ×63. In contrast, WT mice displayed normal neutrophil maturation. **(D)** Enumeration of the number of band neutrophils in the peripheral blood of *Lcn2*^−/−^ mice. The data are presented as mean band cell numbers per 100 leukocytes. Error bars depict SEM. Results are expressed as means ± SEM. *P* < 0.05 was considered statistically significant. *P < 0.05, and **P < 0.01.

The above results showed that Lcn2 deficiency could promote the growth of *E. coli* O157:H7, which indicated that Lcn2 might be involved in the innate immune response to bacterial infection. Previous studies showed that Lcn2 could limit bacterial growth by sequestering the iron-laden siderophore (9, 13). Lcn2 has also been shown to be dramatically upregulated in various inflammatory conditions and is considered as an acute-phase protein (12). Herein, we determined whether a deficiency of Lcn2 has consequences for neutrophil development. We first evaluated the hematological parameters of peripheral blood of *Lcn2*^−/−^ and WT mice. The results showed that the numbers of white blood cells, monocytes, and eosinophils were significantly decreased in *Lcn2*^−/−^ mice (Figure 3A). In contrast, lymphocyte and thrombocyte counts remained unchanged. Furthermore, a granulocyte-specific marker (Ly6G, clone 1A8) was detected using flow cytometry to confirm a decrease of neutrophils in the peripheral blood of *Lcn2*^−/−^ mice (Figure 3B). In addition, Wright-Giemsa staining of peripheral blood smears showed that *Lcn2*^−/−^ neutrophils had atypical bilobed nuclei (band cells), whereas neutrophils of WT mice bore all of the characteristics of mature cells, including ring-shaped segmented nuclei and pale abundant cytoplasm (Figure 3C). Furthermore, the number of circulating band cells in *Lcn2*^−/−^ mice is around 3%, while WT mice had only 1% (Figure 3D).

### Lcn2 Deficiency Reduces the Migration of Neutrophils

**Figure 4:**
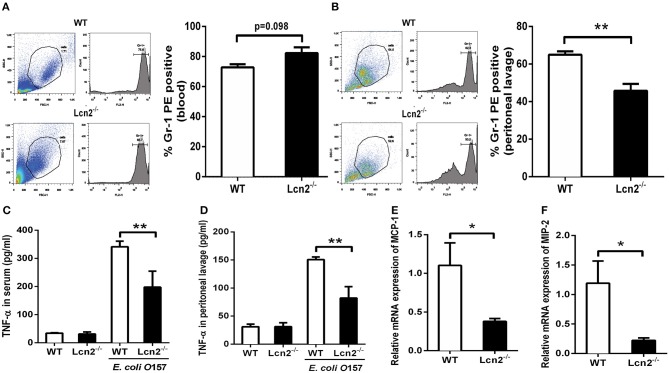
Reduced migration of lipocalin 2-deficient (*Lcn2*^−/−^) neutrophils. **(A,B)** Flow cytometry analysis of peripheral blood and peritoneal exudates of heat-killed *E. coli* O157:H7-challenged mice following staining with a Gr-1 PE Ab. **(C,D)** ELISA analysis of TNF-α in the serum and peritoneal exudates of heat-killed *E. coli* O157:H7-challenged mice. **(E,F)** Quantitative determination of chemokines MCP-1 and MIP-2 mRNA expression in the liver of heat-killed *E. coli* O157:H7-challenged mice. Values are average means of triplicate experiments with two mice per genotype per experiment. Error bars depict SEM. Results are expressed as means ± SEM. *P* < 0.05 was considered statistically significant. *P < 0.05 and **P < 0.01.

Since Lcn2 deficiency can affect the homeostasis of immune cells and the maturation of neutrophils, we speculated that Lcn2 might play an effect on other functions of neutrophils, such as chemotaxis and migration. Normally, the neutrophils from compartments of the bone marrow, peripheral blood, and extravascular space are in dynamic equilibrium (14). Under inflammatory conditions, neutrophils extravasate from the blood compartment to the sites of inflammation (21). We then analyzed neutrophil kinetics in peripheral blood and peritoneum by flow cytometry following Gr-1 staining. Leukocyte extravasation into the peritoneal cavity was studied in a mouse model of inflammation induced by intraperitoneal injection with heat-killed *E. coli* O157:H7 in WT and *Lcn2*^−/−^ mice. Flow cytometry analysis of blood showed that the percentage of neutrophils in the peripheral blood had no significant difference (*P* = 0.098) between WT and *Lcn2*^−/−^ mice (Figure 4A). In contrast, the percentage of extravasated neutrophils in challenged *Lcn2*^−/−^ mice was 30% lower (*P* < 0.01) than that observed in challenged WT mice (Figure 4B). Thus, the results suggested that the migration of neutrophils from the blood into the peritoneum in *Lcn2*^−/−^ mice was reduced. On the other hand, the release of chemokines and cytokines initiates the inflammatory response. Proinflammatory cytokines, such as TNF-α, and chemokines, such as MCP-1, are instrumental in eliciting a neutrophil response. The reduced migration of neutrophils may be due in part to the reduced secretion of any of these chemoattractants. To examine this possibility, we quantitated the levels of TNF-α proteins and transcripts of chemokines. After the inoculation with heat-killed *E. coli* O157:H7, TNF-α proteins of WT mice were increased sharply both in serum and in peritoneal lavage as expected (Figures 4C,D). However, the levels of TNF-α from *Lcn2*^−/−^ mice were elevated mildly, which were significantly (*P* < 0.01) lower than those from WT mice. Moreover, the mRNA expressions of murine chemokines MCP-1 and MIP-2 were both decreased in livers of *Lcn2*^−/−^ mice (Figures 4E,F). Thus, the above results showed that Lcn2 deficiency could impair the migration and chemotaxis ability of neutrophils and disturb the normal secretion of inflammatory cytokines under inflammatory conditions.

### Lcn2 Deficiency Represses the Induction of Inflammatory Cytokines by Macrophages

**Figure 5:**
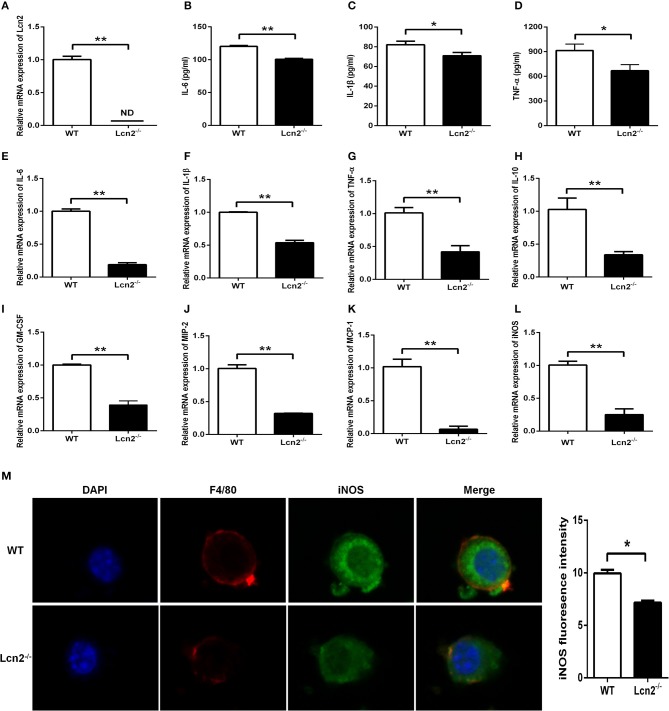
Decreased expression of inflammatory cytokines produced by lipocalin 2-deficient (*Lcn2*^−/−^) macrophages. **(A)** Real-time PCR analysis of Lcn2 mRNA expression levels in the *E. coli* O157:H7-infected primary bone marrow-derived macrophages (BMDMs) from wild-type (WT) and *Lcn2*^−/−^ mice. **(B–D)** ELISA analysis of interleukin (IL)-6, IL-1β, and tumor necrosis factor (TNF)-α levels in the culture medium of the *E. coli* O157:H7-infected BMDMs from WT and *Lcn2*^−/−^ mice. **(E–L)** Real-time PCR analysis of cytokine mRNA expression levels in the *E. coli* O157:H7-infected pBMDMs from WT and *Lcn2*^−/−^ mice. **(M)** The infected BMDMs of WT and *Lcn2*^−/−^ mice were subjected to staining with rabbit monoclonal antibody iNOS, rat monoclonal antibody F4/80, Alexa Fluor 488 goat anti-rabbit IgG, and Alexa Fluor 647 goat anti-rat IgG in blocking buffer (1:200) and observed by fluorescence microscopy. Values are average means of triplicate experiments with two mice used for the isolation of BMDMs per genotype per experiment. Error bars depict SEM. Results are expressed as means ± SEM. *P* < 0.05 was considered statistically significant. *P < 0.05 and **P < 0.01.

Macrophages are the primary producers of cytokines. The observed reduction in cytokines in *Lcn2*^−/−^ mice may also be explained that macrophages need Lcn2 to effectively recognize inflammatory stimuli and to mount an effective cytokine response. To directly test this assumption, we isolated BMDMs from mice treated with *E. coli* O157:H7 and determined the levels of various cytokines. There was no Lcn2 expression in macrophages from *Lcn2*^−/−^ mice (Figure 5A). Secreted inflammatory cytokines including IL-6, IL-1β, and TNF-α were all significantly (*P* < 0.05) decreased in *Lcn2*^−/−^ macrophages compared with WT macrophages (Figures 5B–D). Similarly, the transcripts of inflammatory cytokines, such as IL-6, IL-1β, TNF-α, and IL-10, were all strongly (*P* < 0.01) repressed in *Lcn2*^−/−^ macrophages (Figures 5E–H). The transcript of granulocyte-macrophage colony-stimulating factor (GM-CSF) from *Lcn2*^−/−^ macrophages was also significantly (*P* < 0.01) decreased (Figure 5I), which might explain the reduction of proliferation and differentiation of monocytes. Chemokines are generated at local inflammatory milieu and play an important role in the local recruitment of immune cells. The mRNA levels of chemokines MIP-2 and MCP-1 were both significantly decreased in *Lcn2*^−/−^ macrophages (Figures 5J,K). Macrophages produce inducible nitric oxide synthase (iNOS) that enables the cell to kill pathogens through the production of NO. We further investigated whether deficiency of Lcn2 affected iNOS production. In line with the results of transcription, immunofluorescence analysis showed that iNOS expression was depressed (*P* < 0.05) in *Lcn2*^−/−^ macrophages compared with that of WT macrophages (Figures 5L,M). Thus, the results indicated that Lcn2 played an important role in stimulating the production of these antimicrobial effectors by macrophages and maintained their balances.

### Recombinant Lcn2 Promotes the Migration and Phagocytosis of Macrophages

**Figure 6:**
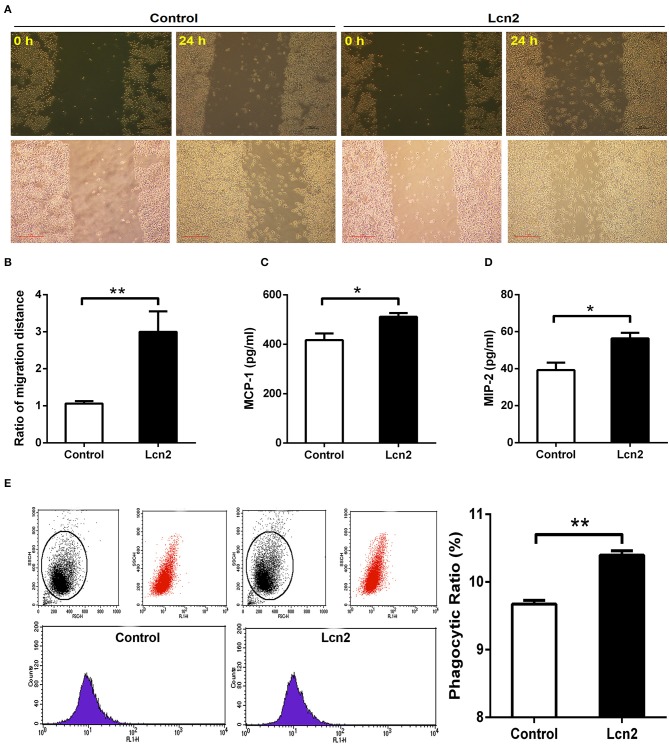
Lipocalin 2 (Lcn2) can promote migration and phagocytosis of macrophages. **(A,B)** Scratch wound healing assay of mouse RAW264.7 macrophages and quantification of the fold change of average migrated distance of cells was measured with microscope (*n* = 3, mean ± SEM, scale bars, 100 μm). **(C,D)** ELISA analysis of monocyte chemoattractant protein (MCP)-1 and macrophage inflammatory protein (MIP)-2 levels in the culture medium of Lcn2-treated macrophages. **(E)** Flow cytometry analysis of mouse RAW264.7 macrophages incubated with FITC-dextran. Values are average means of triplicate experiments with two repetitions per treatment per experiment. Error bars depict SEM. *P < 0.05 and **P < 0.01.

Macrophages are professionally motile cells that carry out a variety of roles in immune surveillance. Transendothelial and interstitial motility is an essential aspect of their function as they must be able to move to specific sites upon demand. Since BMDMs from *Lcn2*^−/−^ mice adhered to walls too firmly to move, we used RAW264.7 macrophages to detect the effect of Lcn2 on the migration of macrophages. Scratch wound healing assay showed that the migration distances of macrophages treated with recombinant Lcn2 were significantly (*P* < 0.01) longer than that of control cells (Figures 6A,B). In addition, chemoattractant cytokines MCP-1 and MIP-2 were both elevated significantly (*P* < 0.05) after Lcn2 treatment (Figures 6C,D), which might explain the increase of migration distance of Lcn2-treated macrophages. Moreover, because macrophages are also professional phagocytes and are highly specialized in the removal of dying or dead cells and cellular debris, we then determined the effect of Lcn2 on the phagocytosis of macrophages. Flow cytometry analysis exhibited that Lcn2 could promote the phagocytosis of FITC-Dextran by macrophages (Figure 6E). Above results indicated that Lcn2 was involved in the immunologic function of macrophages, such as migration and phagocytosis.

## Discussion

Lcn2 has been implicated in diverse physiological processes including apoptosis (22), iron trafficking (13), kidney development (12), and innate immunity (14, 23). We herein provide novel evidence that the absence of Lcn2 increased sensitivity to *E. coli* O157:H7 infection by altering neutrophil homeostasis, reducing the migration of neutrophils, and repressing the expression of inflammatory cytokines by macrophages. Additionally, Lcn2 can also promote the migration and phagocytosis of macrophages.

Lcn2 is considered to be the marker of many inflammatory diseases and involved in various inflammations, including intestinal inflammation, skin inflammation, and metabolic syndrome (24). It is an acute-phase protein known to be highly upregulated during inflammatory conditions (13, 17). In this study, we demonstrated that although bone marrow is the main site of Lcn2 expression normally, Lcn2 was highly induced in almost all detected tissues and mainly induced in the liver of mice in response to *E. coli* O157:H7 infection. This result was in line with that of a previous study (25). We also presented evidence to show that Lcn2 was induced in a parabolic pattern. After intragastric infection, the serum concentration of Lcn2 increased distinctly and then decreased fast. Both mRNA and protein expression showed that Lcn2 peaked by 32 h after challenge. These results are in concordance with observations that Lcn2 may be a marker for inflammation (13, 26). It suggested that strongly induced Lcn2 by *E. coli* O157:H7 infection might play an important role in innate defense against bacterial invasion.

*E. coli* O157:H7 has been a troublesome foodborne intestinal pathogen and involved in numerous human illness outbreaks (27), which requires iron to survive. Although humans or animals contain plenty of iron, the amount available to bacteria may be extremely limited because most iron is bound intracellularly by heme and ferritin, or extracellularly by transferrin and lactoferrin (28). Our previous studies showed that after the infection of *E. coli* K88, iron was inclined to be sequestered within cells and deposited more in tissues rather than serum, which was supposed to restrict iron available to the bacteria (29, 30). To remedy this difficulty, *E. coli* secretes siderophores to remove iron from host iron-binding proteins and transports it into the bacterial cell (29). However, Lcn2 from hosts can specifically bind to siderophores to prevent bacterial uptake of iron. As a result, Lcn2 is bacteriostatic. In this study, we found that there were more bacteria loaded in the blood and liver of *Lcn2*^−/−^ mice, which indicated that *Lcn2*^−/−^ mice succumb to bacterial infection more easily. Furthermore, in a murine model of inflammation with heat-killed *E. coli* O157:H7 infection, we also found that serum Lcn2 was protective against *E. coli* O157:H7. These results proved that elevated Lcn2 after *E. coli* O157:H7 challenge might mediate an innate immune response to inhibit bacterial infection based on iron sequestration.

Lcn2 is a neutrophil gelatinase-associated lipocalin, which is originally isolated from the specific granules of neutrophils (31). We were then wondering whether the knockout of *Lcn2* could affect the balance or development of neutrophils. First, hemocyte analyzer showed that the number of leukocytes, monocytes, and eosinophils in peripheral blood of *Lcn2*^−/−^ mice was significantly lower than that of WT mice. It suggested that Lcn2 deficiency could disturb the normal homeostasis of immune cells in peripheral blood (32). Neutrophils account for a large proportion of leukocytes in the blood (50–70%) and lead the first wave of host defense to infection or tissue damage (33). They were derived from bone marrow hematopoietic stem cells and eventually developed into mature segmented cells through the following process: promyelocytes > myelocytes > meta-myelocytes > band cells > segmented cells (34). In this experiment, more immature band cells in peripheral blood of *Lcn2*^−/−^ mice were observed, which indicated that maturation of neutrophils was impaired in *Lcn2*^−/−^ mice. It is unclear how Lcn2 deficiency contributes to abnormal neutrophil development, but based on our observations, we propose that Lcn2 expression is required for normal neutrophil maturation. Normally, the majority of neutrophils are stored in the hematopoietic cords of bone marrow. Following infection, neutrophils start migrating from the bone marrow to the circulation. The circulating neutrophils then infiltrate from the bloodstream to the inflammatory sites through multiple processes that involve rolling, adhesion, and finally transmigration by passing through the endothelial cells (35). In this study, we also found that the percentage of neutrophils in the peritoneal fluid of challenged *Lcn2*^−/−^ mice was significantly lower than that of WT mice, whereas there was no significant difference in the ration of neutrophils in peripheral blood. This suggested that Lcn2 deficiency could reduce the migration of neutrophils from the blood into peritoneum. Chemoattractant-driven neutrophil migration to the sites of infection and inflammation is a well-coordinated and orderly process (36). In this study, reduced secretions of proinflammatory cytokine TNF-α were found in both serum and peritoneal lavage of *Lcn2*^−/−^ mice. The mRNA expression of chemokines MCP-1 and MIP-2 were also both decreased in livers of *Lcn2*^−/−^ mice. These results indicated that Lcn2 deficiency decelerated neutrophil migration by reducing the expression of some cytokines and chemokines. Lcn2 was reported to act as a central mediator to facilitate the crosstalk between neutrophils and hepatic macrophages via induction of the chemokine receptor CXCR2 (37). It was also found that the adhesion capacity of neutrophils was significantly decreased after Lcn2 deficiency (38). It suggested that the effect of Lcn2 on neutrophil chemotaxis might be related to the expression of adhesion protein and chemokine receptors. Further research is needed to clarify the mechanisms.

Both neutrophils and macrophages provide the first line of defense against invading pathogens. Our results showed that Lcn2 deficiency not only altered neutrophil homeostasis but also reduced their migration. We were then wondering whether Lcn2 deficiency would influence the function of macrophages. In this study, BMDMs were isolated from *Lcn2*^−/−^ and WT mice and stimulated with *E. coli* O157:H7 to establish an inflammatory model. Macrophages monitor the invading pathogens, initiate an inflammatory response, and secrete a large amount of inflammatory factors. The results showed that Lcn2 deficiency resulted in the decreased production of inflammatory cytokines, such as IL-6, IL-1β, and TNF-α. It also downregulated the mRNA levels of all detected factors, including proinflammatory factors, such as IL-6, IL-1β, and TNF-α, anti-inflammatory factor IL-10, and chemokines MCP-1 and MIP-2. Similarly, both protein and mRNA levels of iNOS in BMDM of *Lcn2*^−/−^ mice were also decreased significantly. These results suggested that Lcn2 deficiency could interfere with the normal expression of inflammatory factors secreted by macrophages after *E. coli* O157:H7 stimulation. This finding is similar to the reports that TNF-α is largely inhibited by Lcn2 deficiency in chronic inflammation caused by obesity (39), Lcn2 can induce both IL-6 and IL-10 cytokines during *Brucella abortus* infection (40). However, some studies have also shown that the deficiency of Lcn2 in murine inflammation model caused by lipopolysaccharide (LPS) can significantly increase the expression of proinflammatory factors (41). It was speculated that the difference was based on the way of causing inflammation. Our study focused more on the secretory function of macrophages from *Lcn2*^−/−^ mice instead of the effects of Lcn2 on inflammatory responses. Macrophages can not only stimulate adaptive immunity by secreting cytokines but also kill pathogens and clear up cell debris through phagocytosis (42). Here, we also investigated the effect of Lcn2 on the migration and phagocytosis of RAW264.7 macrophages by treating with recombinant Lcn2. Exogenous Lcn2 could significantly increase the migration and promote the phagocytosis of macrophages, which is conducive to the rapid renovation of tissue damage when inflammation or trauma occurs.

In summary, upon encountering bacteria *E. coli* O157:H7, innate immune cells in most tissues produce and secrete Lcn2 immediately, which is induced in a parabolic pattern and, in turn, limits bacterial growth. Targeted disruption of Lcn2 gene has demonstrated its essential role in the early stages of the innate immune response to bacterial infection. An absence of Lcn2 can lead to an increased susceptibility of mice to *E. coli* O157:H7 infection. It was due to not only the loss of bacteriostatic action of Lcn2 by iron sequestration but also a deficiency of immunological functionality of neutrophils and macrophages. Lcn2 is required for animals to mount a proper host defense to bacterial infection by maintaining normal neutrophil maturation and modulating the function of macrophages.

## Data Availability Statement

The datasets generated for this study are available on request from the corresponding author.

## Ethics Statement

The animal study was reviewed and approved by Animal Care and Use Committee of Zhejiang University.

## Author Contributions

QW and HD designed the research. QW, SL, XT, and FW developed reagents and performed experiments. QW, SL, and HD analyzed the data. QW, SL, LL, and HD wrote the manuscript.

### Conflict of Interest statement

The authors declare that the research was conducted in the absence of any commercial or financial relationships that could be construed as a potential conflict of interest.

## Supplementary Material

Figure S1 Establishment of a mouse model of *E. coli* O157:H7 challenge. Effects of challenge on the morphology of liver, kidney, and jejunum of mice **(A)**, and transcription levels of inflammatory cytokines TNF-α, IL-1β, and IL-10 in the liver **(B)**. 18S rRNA was used as the housekeeping gene. The mRNA expression ratio was normalized to the mean value of control group of 1. Values are average means of triplicate experiments. Error bars depict SEM (*n* = 4). Results are expressed as means ± SEM. ^**^*P* < 0.01.
