# Supplementary figures and images for: Lipocalin 2 Protects Against Escherichia coli Infection by Modulating Neutrophil and Macrophage Function
Source: Front Immunol. 2019 Nov 8;10:2594. doi: 10.3389/fimmu.2019.02594 (PMC6857527; doi:10.3389/fimmu.2019.02594)

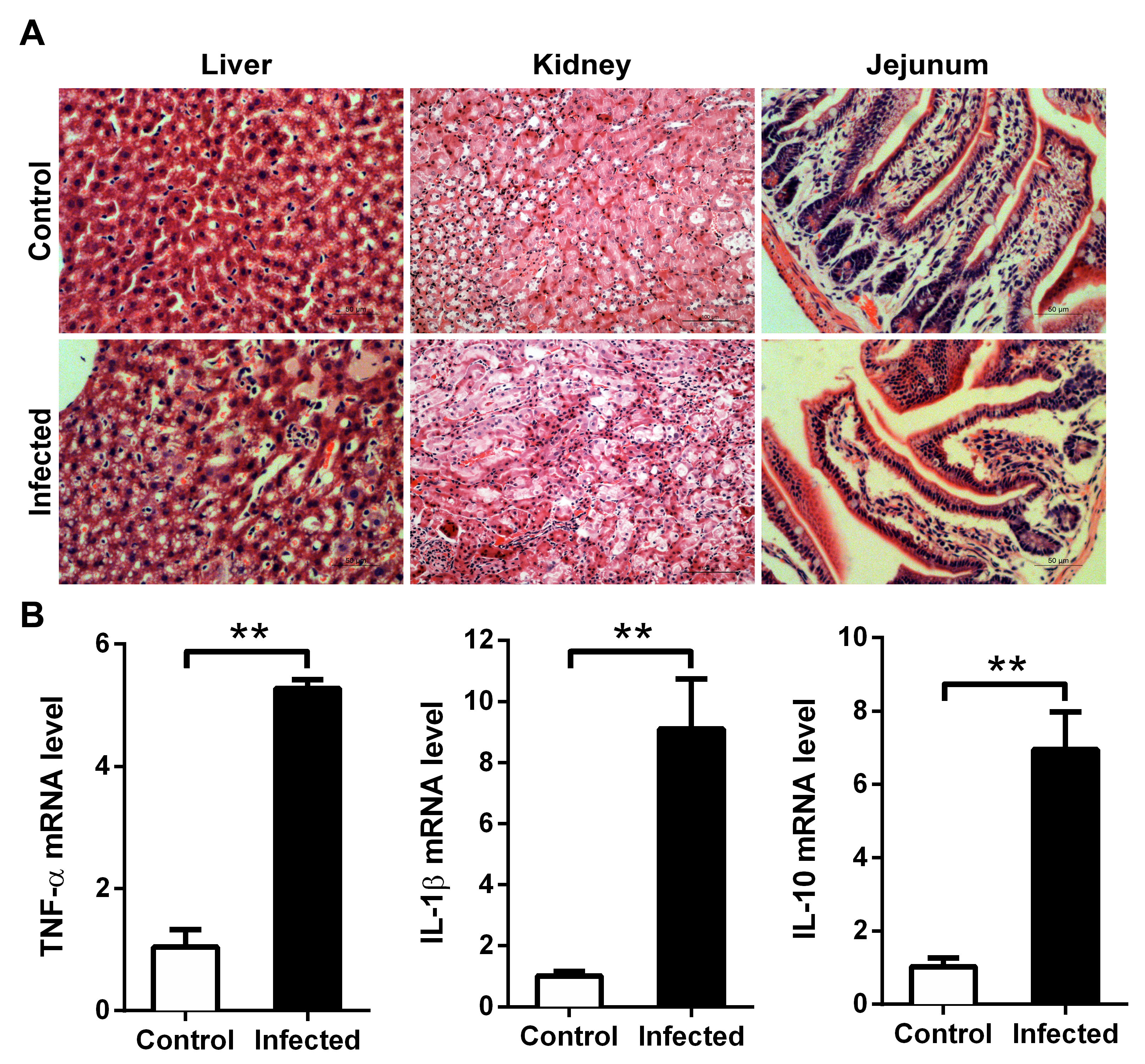

Supplement: Figure S1 — Establishment of a mouse model of E. coli O157:H7 challenge. Effects of challenge on the morphology of liver, kidney, and jejunum of mice (A), and transcription levels of inflammatory cytokines TNF-α, IL-1β, and IL-10 in the liver (B). 18S rRNA was used as the housekeeping gene. The mRNA expression ratio was normalized to the mean value of control group of 1. Values are average means of triplicate experiments. Error bars depict SEM (n = 4). Results are expressed as means ± SEM. **P < 0.01. [file Image_1.TIF]
